# Transdermal Nicotine as a Treatment Option for Ulcerative Colitis: A Review

**DOI:** 10.7759/cureus.11096

**Published:** 2020-10-22

**Authors:** Vishmita Kannichamy, Ishan Antony, Vinayak Mishra, Amit Banerjee, Arohi B Gandhi, Ifrah Kaleem, Josh Alexander, Mohamed Hisbulla, Safeera Khan

**Affiliations:** 1 Internal Medicine, California Institute of Behavioral Neurosciences and Psychology, Fairfield, USA; 2 General Medicine, California Institute of Behavioral Neurosciences and Psychology, Fairfield, USA

**Keywords:** ulcerative colitis (uc), inflammatory bowel disease, nicotine

## Abstract

Ulcerative colitis (UC) is primarily a disease of non-smokers or ex-smokers. Since there have been previous claims of the beneficial effects of transdermal nicotine, researchers studied its efficacy to include it in the treatment regimen: to prevent remissions and as maintenance therapy. This review aims to evaluate the efficacy of transdermal nicotine as a treatment option for mild to moderately active ulcerative colitis. We shortlisted 22 articles after a careful analysis and elimination process. These articles were reviewed and analyzed, and it was found that transdermal nicotine in combination with conventional therapy was more beneficial than individual treatment with either. Further controlled studies evaluating the appropriate dosage for remission and maintenance treatment needs to be done.

## Introduction and background

Ulcerative colitis is a non-transmural inflammatory disease of the colon with episodic flares and remissions along with abdominal pain, diarrhea, and rectal bleeding [1]. The interrelationship between non-smoking and ulcerative colitis was identified and reported by Harries et al. in 1982. He found that only 8% of the patients with the disease were smokers as compared with 44% of matched controls attending a fracture clinic [2]. Numerous case-control studies and meta-analysis have been conducted since then, establishing similar findings. This incidental finding has persuaded many to consider nicotine to be of therapeutic value given transdermally or by enema to treat ulcerative colitis [3]. However, the etiology, pathogenesis, and treatment regimen for the disease remain unclear. Several clinical trials have examined the potency of nicotine in the form of enemas, transdermal patches, and chewing gum in the remissions of ulcerative colitis and have stated that nicotine was an effective treatment option.

In contrast, some studies reported no efficacy of nicotine in the remission of ulcerative colitis [4-5]. Genetic factors and how they interact with tobacco smoke to modify the risk of inflammatory disease are currently under study [6]. This finding of relevance between smoking with ulcerative colitis has been the subject of scrutiny in the hope that it may help identify alternative therapeutic options and pathogenic mechanisms involved in the disease. This review focuses on the efficacy of transdermal nicotine as a treatment option for ulcerative colitis.

## Review

Methods

We searched electronic databases (i.e., PubMed, PMC, Google Scholar, and MEDLINE) since the inception of the topic until September 2017. Keywords such as 'ulcerative colitis' and 'smoking' or "nicotine' were used separately and in combination. Also, MeSH search terms' ulcerative colitis' and 'nicotine' were used to extract all the relevant data. We included different study types, including observational studies, review articles, and randomized control trials (RCTs). No gray literature was included. The patients with active UC and those with UC in remission were the population group included. The articles with full free text and abstract were included, with the English language restriction applied. After removing duplicate articles and enforcing quality checks, 22 relevant articles were identified through manual review. The reference list was reviewed to include relevant articles. Table 1 shows the results of the keyword searches.

**Table 1:** Results of keyword searches

keywords	PubMed	PubMed Central	Google Scholar
Ulcerative Colitis	9,400	58,957	6,46,000
Nicotine	10,547	78,812	11,30,000
Ulcerative Colitis and Nicotine	52	1166	9,940

Inclusion and exclusion criteria

All papers included were written in the English language and included data collected and reviewed from inception till 2020. The included scientific papers contain data collected from various sample sizes in a particular geographical area. Only studies conducted on humans were included; animal studies were excluded. All the articles included patients who previously suffered from, are currently diagnosed with, or have been remitted with Ulcerative Colitis. This review excluded patients with other inflammatory bowel diseases like Crohn's disease. The results of these studies were statistically analyzed values with estimates of sensitivity; specificity did manually.

Results

The screening process identified 159 studies after typing in the keywords in electronic databases and using the MeSH strategy. After the application of inclusion and exclusion criteria, we were left with 51 articles. With further removal of duplicate articles, 22 articles were shortlisted. Out of the 22, four are review articles, seven clinical trials, four randomized control trials, one comparative study, and six observational studies. We identified a total of 308,273 patients for review, which included both patients with active ulcerative colitis and those with ulcerative colitis in remission. In all the 23 articles, it was confirmed that there is a positive association between nicotine and ulcerative colitis. Most of the trials were conducted for six weeks to 12 weeks, and observational studies were done for up to 26 weeks.

Discussion

Proposed Mechanisms in the Influence of Nicotine on Ulcerative Colitis

Due to the unknown etiology of ulcerative colitis, it has become challenging to explain the undoubted association between nicotine and ulcerative colitis. Various studies have indulged in answering this question. Many mechanisms have been suggested. However, none have been entirely satisfactory.

It has been noted that nitric oxide (NO) has an effect on colonic epithelial cell function, but this remains to be confirmed [7]. Another possible theory is that nicotine influences cellular and humoral immunity by decreasing all the immunoglobulin (Ig) levels except IgE. Their levels are increased, thereby mimicking the actions of immunosuppressive drugs, perhaps through the stimulation of endogenous steroid release [8-9]. Nicotine has also been known to cause a prothrombic state by directly inhibiting vascular prostacyclin (PGI2) secretion or by elevating plasma fibrinogen levels, increasing plasma viscosity, or increasing packed cell volume. This eventually leads to a combination of decreased rectal blood flow and hypercoagulability, causing exacerbations of the condition. Hence, this cannot explain the protective effect of nicotine on UC [8].

Nicotine has also been found to suppress the in vivo Th2 cell function as measured by IL-10 production. Nicotine can affect gut motility, but its relevance is currently not fully understood [8]. Some of the other mechanisms under study include the effect of endogenous glucocorticoids, antioxidant and oxygen free radicals, and their relationship with ulcerative colitis. Table 2 comprises all the studies discussing the mechanism of action of nicotine.

**Table 2:** Studies discussing proposed mechanisms in the influence of nicotine on ulcerative colitis

Author and Year of Publication	Type of Study	Number of Patients	Mechanism Stated	Conclusion/Result
Lunney P C et al., 2012 [10]	Review	374	Interferes with cellular and humoral immunity eicosanoid mediated inflammation. Antioxidant and oxygen-free radicals, Gut permeability, Endogenous glucocorticoid, Colonic mucus, Mucosal blood flow, Thrombosis, and Gut permeability	Further studies on the minimum therapeutic dose and adverse effects need to be studied before nicotine can be used along with conventional therapy.
Osborne M J et al.,1992 [8]	Review	1256	Changes in the permeability of the intestine to harmful substances, immunosuppression, and smoking have been shown to alter both cellular and humoral immunity, Abnormal colonic mucus, A tendency to arterial thrombosis	Association is definite seems, however, beyond dispute.
Richardson et al. [11]	Observational study	160	Nitric oxide (NO) medicated mechanisms are involved.	Decreased expression of α3 nAChRs in the epithelium of patients with ulcerative colitis, a finding seen in both smokers and non‐smokers alike.
Guslandi M et al.,1999 [12]	Observational study	16	Alterations in the cellular and humoral immune responses. Nicotine interferes with the inflammatory response, Decreases intestinal motility, Reduction in intestinal blood flow.	Nicotine appears to be ineffective in treating clinical relapses. In contrast, smoking seems to protect otherwise healthy individuals from UC. Safer nicotine preparations will prove to be useful for clinical use.
Green J T et al., 2000 [3]	Clinical trial	27	Nitric oxide mediates a therapeutic effect of nicotine in UC.	Nicotine reduces circular muscle activity, predominantly through the release of nitric oxide-this appears to be 'up-regulated' in active ulcerative colitis
Thomas G A et al., 2000 [13]	Review	230	Immune system Inflammatory cascade, Gut motility, Mucus production, Gut permeability, Gut blood flow, Platelet activation	Transdermal nicotine may be beneficial for patients with active UC, but different delivery systems and doses need to be explored more.
Madretsma S et al., 1996 [14]	Observational study	11	Nicotine is known to suppress in-vivo production of Th2 cell function.	Nicotine in vivo has an inhibitory effect on Th2 cell function as measured by the inhibition of IL-10 production but does not appear to have any effect on Th1 cell function.

Tolerability and Efficacy of Transdermal Nicotine

Adverse effects were more significant during therapy with transdermal nicotine patches in all three RCTs considered [15-17]. The side effects most commonly reported were nausea, lightheadedness, tremors, headache and sleep disturbances, and skin irritation. The side effects seen were significantly higher than prednisolone or mesalamine therapy, but it must be noted that those taking glucocorticoids were included in different trial groups [12,18].

In general, adverse reactions took place much more often in lifelong non-smokers than in former smokers. No clear correlation was established between nicotine plasma levels and the incidence of adverse effects.

In conclusion, transdermal nicotine treatment resulted in frequent adverse effects leading to non-compliance, but most patients were able to complete therapy. Most of the studies expressed that transdermal nicotine patches alone were no better than placebo but, in combination with conventional therapy, proved to be beneficial. Table 3 is a compilation of all the studies discussing the tolerability of transdermal nicotine.

**Table 3:** Studies discussing the tolerability of transdermal nicotine

Author and Year of Publication	Type of Study	Number of Patients	Comparator	Study Length	Conclusion/Result
Lunney P C et al., 2012 [10]	Review	374 (72)	placebo	Six weeks	The concentrations of nicotine and cotinine in the blood were lower than expected throughout the study in patients of the nicotine group, suggesting poor compliance to therapy.
Pullan R D et al., 1994 [17]	Randomized control trial	72	placebo	Six weeks	The addition of transdermal nicotine to conventional maintenance therapy improves symptoms in patients with ulcerative colitis.
Sandborn W J et al., 1997 [16]	Randomized control trial	64	Placebo	Four weeks	Highest tolerated dosage, i.e. 22 g/d or more for four weeks, is considered best for controlling the manifestations of the disease.
Richardson C E et al, 2003 [11]	Observational Study	160 (43)	placebo	26 weeks	The intensity or distribution of epithelial staining did not vary before and after treatment with a nicotine patch.

Nicotine Patch Vs. Conventional Therapy

A study conducted by Thomas G A et al. included 61 patients with active ulcerative colitis [15]. The patients were treated with either an incremental dosage of transdermal patches (between 15 and 25 mg daily) or 15 mg of prednisolone for six weeks. Patients taking topical were asked to discontinue their medication at the beginning of the study and those taking mesalazine on day 10. Only 43 patients completed the study. Out of 19 in the nicotine group, six achieved full sigmoidoscopic remission compared to the 14 out of 24 in the prednisolone group. Therefore, they concluded that nicotine alone offered minimal benefit and was not as effective as prednisolone.

In contrast, Guslandi M et al. conducted a similar study in 1998 where patients with mild to moderate UC relapse during maintenance treatment (mesalamine 1 g b.i.d.) were given either transdermal nicotine or prednisone for five weeks [19]. The results showed that relapses occurred earlier and to many patients in the prednisolone group (60%) than those treated with nicotine (20%). This study showed that remissions induced by nicotine last longer than oral corticosteroids and that nicotine proves useful in treating mild to moderately active UC.

In another trial conducted by Guslandi M et al., they concluded that nicotine patches proved to be an excellent alternative to steroids in patients with mild to moderate UC after four weeks of treatment with transdermal nicotine (15 mg daily) along with conventional treatment (mesalazine 1 g t.i.d.) [19].

It is clear from the above data that transdermal nicotine alone has limited efficacy in the treatment. If administered in combination with mesalazine or prednisolone, it will give superior results. Table 4 is a compilation of all the studies comparing the efficacy of transdermal nicotine with conventional therapy.

**Table 4:** Studies comparing the efficacy of transdermal nicotine with conventional therapy

Author and Year of Publication	Type of Study	Number of Patients	Comparator	Study Length	Conclusion/Result
Guslandi M et al., 1999 [12]	Observational study	16	Prednisolone	Four weeks	A short period could have hampered a proper histological outcome, but no sigmoidoscopic improvements were seen.
Thomas G A et al., 1996 [18]	Clinical trial	61	Prednisolone	Six weeks	Prednisolone was seen to be more effective.
Guslandi M et al., 1998 [19]	Randomized control trial	-	Prednisolone	Five weeks	Nicotine is useful in UC with mild or moderate activity and remissions induced by it last longer.
Guslandi M et al.,1996 [20]	Randomized control trial	10	Mesalazine	Four weeks	Nicotine patches prove to be a good alternative to steroids in patients with mild to moderate UC.

## Conclusions

This review aimed to determine whether transdermal nicotine can be used as part of the treatment regimen for a patient with mild to moderately active ulcerative colitis. After analysis, it can be understood that transdermal nicotine alone proved to be of little value; instead, nicotine patches, in combination with traditional treatment, have offered more significant benefits. The available treatment options for ulcerative colitis is limited and far from satisfactory. The options include corticosteroids and mesalazine, the former being useful for treating acute colitis, but its use is limited in the maintenance of clinical remissions. The difficulty in the treatment of UC sources from its unknown etiology, this study mainly discusses all the possible mechanisms through which the disease might operate. The study also reviews the tolerability of nicotine formulations and their efficacy in treating the disease. The limitations were mainly the sample population. No specific age group was focused on. Limited randomized control trials and clinical trials were available. In addition, the availability of recent studies on this topic was scarce. After careful evaluation, it is safe to assume that transdermal nicotine, in combination with mesalazine or glucocorticoids, has been more effective than individual use in preventing clinical remissions, but the dosage tolerability and dosage most effective for treatment have to be further studied for accurate recommendations. More controlled clinical trials are required to provide a therapeutic solution for UC.
